# An Analysis Between Body Composition and Vertigo Post-Coronavirus Disease 2019 Patients

**DOI:** 10.1055/s-0044-1791729

**Published:** 2025-04-15

**Authors:** Luciana Lozza de Moraes Marchiori, Glória de Moraes Marchiori, Licia Sayuri Tanaka Okamura, Daiane Soares de Almeida Ciquinato, Braulio Henrique Magnani Branco

**Affiliations:** 1 Interdisciplinary Health Promotion Intervention Laboratory (LIIPS), Centro Universitário Cesumar (UniCesumar), Maringá, PR, Brazil; 2 Research Group on Physical Education, Physiotherapy, Sports, Nutrition and Performance, Centro Universitário Cesumar (UniCesumar), Maringá, PR, Brazil; 3 Departament of Occupational Health, Prefeitura de Cambé, Cambé, PR, Brazil; 4 Postgraduation Program in Health Promotion, Centro Universitário Cesumar (UniCesumar), Maringá, PR, Brazil

**Keywords:** vertigo, dizziness, body composition, COVID-19, long COVID

## Abstract

**Introduction** An association between the sensation of vertigo, and body composition has not been investigated in post-coronavirus disease 2019 (COVID-19) patients.

**Objective** To evaluate the probable association between the sensation of vertigo and body composition—as amount of fat, bone, and muscle—in post-COVID-19 patients.

**Methods** Cross-sectional study with a sample of post-COVID-19 patients who responded to the visual analog scale (VAS) and the Dizziness Handicap Inventory (DHI). Clinical assessment and electrical bioimpedance were used to determine body composition.

**Results** There were 105 participants evaluated, 61% (n = 64) of whom were male, aged 49.5 ± 11.7 years, with an average height of 165.6 ± 19.9 cm, body weight of 87.6 ± 20 kg, and body mass index (BMI) of 31.1 ± 5.4 kg/m. The prevalence of self-reported vertigo was 51.4% (n = 54); of these, 11.4% (n = 12) related vertigo before the diagnosis of COVID-19, and 40% (n = 42) related vertigo during or after COVID-19. Furthermore, 37.2% (n = 39) of the sample reported that vertigo persisted after medical discharge. In the comparative analysis, the data showed a significant difference between the groups with and without vertigo for height ( *p* = 0.001), body mass ( *p* = 0.006), body water ( *p* = 0.001), lean mass ( *p* = 0.002), fat-free mass ( *p* = 0.001), and musculoskeletal mass ( *p* = 0.001).

**Conclusion** There was a significant association between body composition and the complaint of vertigo in post-COVID-19 patients, suggesting that these aspects should be considered when assessing and can contribute to the construction of knowledge about long COVID.

## Introduction

The coronavirus disease 2019 (COVID-19), caused by the severe acute respiratory syndrome coronavirus 2 (SARS-CoV-2) virus, can lead to deleterious effects and symptoms known as post-COVID syndrome, which include musculoskeletal changes and neurotological symptoms such as balance disorders. 1 2 In addition, immobility associated with prolonged hospitalization in patients with COVID-19 may contribute to decrease in muscle mass, which could potentially make them more susceptible to the development of myalgia, neuropathies, and balance deficits such as vertigo. 2 3 4

A loss of muscle mass, quality, and function, which is particularly evident in the respiratory muscles, termed sarcopenia, has been associated with many adverse clinical outcomes. 5 6 Muscle density has been associated with successful extubation and inversely associated with the number of ICU complications, length of stay, and hospital mortality. 4 7 The present study aimed to evaluate the body composition and cardiorespiratory fitness of overweight or obese individuals after COVID-19. Hospitalized individuals had significantly higher values for fat mass and body fat percentage than those who had not been hospitalized. 7

Dizziness is a general, non-specific term to indicate a sense of disorientation. Vertigo is a subtype of dizziness and refers to an erroneous perception of self- or object-motion or an unpleasant distortion of static gravitational orientation that is a result of a mismatch between the vestibular, visual, and somatosensory systems. 8 It has a multifactorial etiology; however, treatment is usually successful if the specific cause can be identified. 8 9 Common causes can be revealed by systematic clinical examination and selective laboratory tests. 7 8 9 The more common causes of vertigo, dizziness, and imbalance are sensory deficits, such as bilateral vestibular hypofunction, polyneuropathy, and impaired visual acuity; benign paroxysmal positional vertigo; and central disorders such as cerebellar ataxia and normal-pressure hydrocephalus, but further relevant factors include sedative or antihypertensive medication and loss of muscle mass. 7 8 9 10

Clinical manifestations after COVID-19 include symptoms of vertigo and dizziness, which is rather not surprising, since SARS-CoV-2 neurotropism may inflict a broad spectrum of neuropathic effects. 11 The widespread nature of central and peripheral audiovestibular pathways suggests that there may be several probable pathophysiological mechanisms that can lead to symptoms of vertigo and dizziness. 11 During the COVID-19 pandemic most cases of vestibular disorders were attributable to psychological causes exacerbated by stress following acute infection and mandatory quarantine. A study assessed the prevalence and characteristics of dizziness and vertigo among patients with mild-to-moderate COVID-19; it included 1,512 subjects (765 females and 747 males), with a median age of 51 ± 18.4 years. New-onset dizziness was reported by 251 (16.6%) patients, among whom 110 (43.8%) complained of lightheadedness, 70 (27.9%) of disequilibrium, 41 (16.3%) of presyncope, and 30 (12%) of vertigo. This same study analyzed in detail the prevalence and pathophysiological mechanisms of the different types of balance disorders in a large sample, and the results suggest that dizziness should be included among the main symptoms of COVID-19 because 1/6 of patients reported this symptom, with females being significantly more affected than males (20.3 vs 12.9%, *p* < 0.001). Most cases of dizziness were attributable to lightheadedness, which was probably exacerbated by psychophysical stress following acute infection and mandatory quarantine. 11

A limited number of studies have examined body composition in patients with vertigo; among these, only a few have observed the association between body composition and vertigo in post-COVID patients. Thus, the present study aimed to verify the existence of a possible association between the sensation of vertigo and body composition in patients post COVID-19.

## Methods

The present cross-sectional study is part of broader research named “Post COVID Project”. The Human Research Ethics Committee of the Centro Universitário de Maringá (UniCesumar) approved the project (CAAE: 39056920.0.0000.5539). Before beginning the study, all patients were informed about its objectives and procedures and signed an informed consent form (ICF). The inclusion criteria for this study were as follows: being 19 to 65 years old; having been positively diagnosed with COVID-19 via qualitative molecular test (reverse transcription-polymerase chain reaction [RT-PCR]), presenting the test result and/or hospital discharge after COVID-19 treatment; having contracted COVID-19 between March 1 and July 1, 2021; having received the first dose of the COVID-19 vaccine; and having a medical report allowing them to undergo cardiorespiratory fitness tests. Based on the exclusion criteria, the study did not accept patients with debilitating neurological diseases or mobility difficulties (those who needed a walking stick or wheelchair).

Data were collected between August and December 2021 by trained assessors of the multi-professional team at the institution's Interdisciplinary Health Promotion Intervention Laboratory (LIIPS/UniCesumar), guided by physicians, physical therapists, speech-language-hearing therapists, nutritionists, and physical educators. Patients were recruited via referral from the municipal hospital, after their discharge. In order to carry out the research, the participants' clinical information was collected.

On their first visit to the research laboratory, participants were clinically assessed (arterial pressure, glycemia, oxygen saturation, physical assessment with anthropometry, body composition with electrical bioimpedance, and cardiorespiratory effort test) and answered a standardized questionnaire with 90 open-ended and closed-ended items, encompassing data on medical history, preexisting diseases (self-reported hypertension and diabetes, obesity, hyper or hypothyroidism, and hypo or hypercholesterolemia), need for hospitalization, length and type of hospital stay, symptomatology (tinnitus, dizziness, vertigo, aural fullness, hearing loss, headache, anosmia, ageusia) during and/or after COVID-19, and duration of symptoms after hospital discharge. Patients also underwent hearing assessments on their second visit and blood tests on the third one. In the auditory evaluation, the first procedure was the inspection of the external auditory canal. Subsequently, the participants answered the questions of the audiological anamnesis and were evaluated by the preliminary tonal and logo audiometries (Speech Reception Threshold [SRT] and Speech Intelligibility Index [SII]). The study used data collected on their first visit to the research laboratory.

Then, their body composition was assessed, and their anthropometric measures were taken, measuring their body weight and height, and calculating and classifying the body mass index (BMI). Patients whose BMI ranged from 18.00 to 24.99 kg/m ^2^ were classified as normal weight; those from 25.00 to 29.99 kg/m2 as overweight; and those 30.00 or more kg/m ^2^ as obese. 7 8 10 11 12

The vertigo-related questions asked specifically about feeling vertigo sensation at the time of the assessment. They were also asked about symptom type and duration, and its relationship with COVID-19 to verify whether it was previous or posterior to it, or whether it appeared during the COVID-19 infection. The Dizziness Handicap Inventory (DHI), in its Brazilian Portuguese version, was used in patients who reported dizziness during the initial questionnaire. It constitutes a self-assessment instrument with 25 items to verify the functional, emotional, physical, and functional repercussions caused by dizziness on quality of life. 13 The visual analog scale (VAS) was used to verify the degree of discomfort in patients who reported dizziness during the application of the initial questionnaire. On this scale, with the help of an appropriate colored ruler, the patient assigns a score from 0 to 10 to dizziness, with a score of 10 referring to the greatest discomfort. 14

Statistical data analysis was conducted using the IBM SPSS Statistics for Windows, version 20.0 (IBM Corp., Armonk, NY, USA), with a 95% confidence interval (CI) and 5% significance level ( *p* < 0.05). The parametric distribution of the data was verified using the Shapiro-Wilk test with no assumption of normality, whereas the Mann-Whitney test was used for continuous variables. The Mann–Whitney effect size was also calculated with the following equation: r = Z /√n, in which “r” is the correlation coefficient, “Z” is the standardized U-value, and “n” is the number of observations. The r-value and Pearson correlation coefficient (r) coincided. 15 The association with categorical variables was analyzed using the Chi-squared test. The Phi and Cramer V values were also calculated. The statistical power of the sample was calculated post hoc in the GPower 3.1.7 software, using the mean and standard deviation for the musculoskeletal mass of the group without vertigo (32.5 ± 7.7 kg; n = 51), and of the vertigo group (27.6 ± 6.5 kg; n = 54), two tail, and α = 0.05, with a power of 93%.

## Results

A total of 105 participants who participated in the evaluation and answered the vertigo questionnaires were evaluated, 61% (n = 64) of the sample were male and 39% (n = 41) female, who were aged 49.5 ± 11.7 years, with an average body mass of 87.7 ± 19.7 kg, height of 165.6 ± 19.9 cm, body weight of 87.6 ± 20 kg, BMI of 31.1 ± 5.4 kg/m ^2^ , total body water of 39.8 ± 9.3 L, lean mass of 50.7 ± 12.3 kg, fat-free mass of 53.9 ± 12.6 kg, musculoskeletal mass of 29.9 ± 7.4 kg, fat mass of 33.4 ± 12.6 kg, and fat percentage of 37.7 ± 9.0%. The prevalence of self-reported vertigo was 51.4% (n = 54); of these, 11.4% (n = 12) related vertigo before the diagnosis of COVID-19 and 40% (n = 42) related vertigo during or after COVID-19. Furthermore, 37.2% (n = 39) reported that vertigo persisted after medical discharge.

The Chi-squared test, according to Table 1 , showed an association between vertigo and gender ( *p* = 0.006), with women presenting a higher percentage of vertigo (68.3%); an association was also observed between vertigo and the feeling of restful sleep ( *p* = 0.004); the group with vertigo reported non-restorative sleep more frequently (68.2%); additionally, an association was observed between vertigo and fatigue, in all classifications ( *p* = 0.024); finally, an association was also detected between vertigo and the length of hospital stay ( *p* = 0.044); for more information see Table 1 .

**Table 1:** Samplés descriptive data (n = 105)

Categorical Variables	No Vertigo (n = 51; 48%) n (%)	Vertigo (n = 54; 51.4%) n (%)	*p* -value chi-squared
**Sex**			
Males	38 (59.4)	26 (40.6)	0.006*
Females	13 (31.7)	28 (68.3)	
**Age range**			
24–44 years	19 (50)	19 (50)	
45–64 years	30 (50)	30 (30)	0.609
≥ 65 years	2 (28.6)	5 (71.4)	
**Sleep quality**			
Good sleep quality	37 (60.7)	24 (39.3)	0.004*
Poor sleep quality	14 (31.8)	30 (68.2)	
**Fatigue**			
N/A	12 (85.7)	2 (14.3)	
small efforts	18 (45)	22 (55)	0.024*
medium efforts	17 (43.6)	22 (56.4)	
great efforts	4 (33.3)	8 (66.7)	
**Oxygen therapy**			
No	9 (42.9)	12 (57.1)	0.558
Yes	42 (50)	50 (50)	
**Oxygen type**			
N/A	9 (40.9)	13 (59.1)	
Catheter	14 (46.7)	16 (53.3)	0.769
Oxygen mask	7 (58.3)	5 (41.7)	
Both (catheter and oxygen mask)	21 (51.2)	20 (48.8	
**Time of use of oxygen therapy**			
N/A	10 (43.5)	13 (56.5)	
up to 5 days	13 (39.4)	20 (60.6)	0.247
6 days or more	28 (57.1)	21 (42.9)	
**Need for hospitalization**			
No	4 (57.1)	3 (42.9)	0.711
yes	47 (48)	51 (52)	
**Type of hospitalization**			
N/A	4 (57.1)	3 (42.9)	
Nursery	22 (37.9)	36 (62.1)	0.055
ICU	10 (76.9)	3 (23.1)	
Both (nursery/ICU)	15 (55.6)	12 (44.4)	
**Days of hospitalization in the ward**			
N/A	11 (64.7)	6 (35.3)	
< 7 days	13 (33.3)	26 (66.7)	0.044*
≥ 8 days	27 (51.1)	22 (44.9)	
**ICU days with non-invasive ventilation**			
N/A	27 (44.3)	34 (55.7)	
< 7 days	11 (55)	9 (45)	0.581
≥ 8 days	13 (54.2)	11 (45.8)	
**days with invasive ventilation**			
N/A	29 (42.6)	39 (57.4)	
< 7 days	6 (46.2)	7 (53.8)	0.127
≥ 8 days	16 (66.7)	8 (33.3)	
**Vertigo persistence time**			
did not persist	−	15 (27.8)	
< 2 months	−	18 (33.3)	−
2–4 months	−	11 (20.4)	−
> 6 months	−	10 (18.5)	−
**Dizziness Handicap Inventory** (DHI)			
DHI-fu	−	8 [4–10] ^a^	−
DHI-em	−	4 [4–8]	−
DHI-fis	−	4 [2–6]	−
DHI-total	−	16 [10–24]	−
**VAS Vertigo**	−	6 [5–8]	−

Abbreviations: DHI, Dizziness Handicap Inventory; DHI-em, DHI – emotional domain; DHI-fis, DHI – physical domain; DHI-fu, DHI – functional domain; DHI-total, total sum of DHI; ICU, intensive care unit; N/A, not applicable; VAS, visual analog scale.

Note: ^a^ , median and interquartile range (25–75%)

In the comparative analysis, the data showed a significant difference between the groups with and without vertigo for height ( *p* = 0.001), body mass ( *p* = 0.006), body water ( *p* = 0.001), lean mass ( *p* = 0.002), fat free mass ( *p* = 0.001), musculoskeletal mass ( *p* = 0.001), but not for fat mass ( *p* = 0.624), fat percentage ( *p* = 0.155), and BMI ( *p* = 0.791); The group with vertigo had lower musculoskeletal mass when compared to the others ( *p* < 0.05). The effect size was small. These data are presented in Table 2 .

**Table 2:** Comparative analyses between the groups with/without vertigo and body composition (n = 105)

variables	No Vertigo (n = 51)	Vertigo (n = 54)	p-value (Mann-Whitney)
age (years)	47 [39–59] ^a^	53 [41–58]	0.339
height (cm)	172 [164–181]	162 [156–172]	0.001*; 0.03 ^b^
Weight (kg)	89.3 [78.5–98.6]	81.9 [71.7–89.1]	0.006*; 0.02 ^b^
IMC	30.6 [27.5–33.9]	29.7 [27.5–33.5]	0.791
ACT (l)	41.7 [36.6–49.5]	36 [30.9–41.7]	0.001*; 0.03 ^b^
MM (kg)	53.4 [43.3–63.6]	46.2 [39.7–53.4]	0.002*; 0.03 ^b^
MLG	56.6 [49.6–67.4]	48.9 [41.9–55.5]	0.001*; 0.03 ^b^
MME (kg)	31.6 [26.9–38.2]	27.1 [22.7–31.2]	0.001*; 0.03 ^b^
MG (kg)	34.5 [23.6–43.1]	30.6 [25.7–38.4]	0.624
% GORD	35.6 [27.8–43.8]	40 [33.4–63.4]	0.155

Abbreviations: % FAT, percentage of fat; ACT, total body water; BMI, body mass index; FFM, fat free mass; MM, lean mass; MME, skeletal muscle mass; MG, fat mass.

Notes: ^a^ , median and interquartile range (25–75%); *, statistically significant; ^b^ , effect size;

No significant correlations were observed for the musculoskeletal mass, VAS, and DHI questionnaire domains, as well as for the fat mass, VAS, and DHI domains ( *p* > 0.05). For the other investigated variables, no significant correlations were observed ( *p* > 0.05). ( Table 3 )

**Table 3:** Correlation between VAS, DHI and corporal composition

Variables	Spearman Correlation Coefficient (r _s_ )	*p* -value
Height X VAS	−0.135	0.394
Height x DHI-fu	−0.007	0.961
Height x DHI-em	−0.021	0.881
Height x DHI-fis	−0.016	0.911
Height x DHI-total	−0.042	0.761
Weight X VAS	0.080	0.613
Weight x DHI-fu	0.031	0.826
Weight x DHI-em	0.002	0.989
Weight x DHI-fis	−0.064	0.643
Weight x DHI-total	0.021	0.879
BMI X VAS	0.278	0.075
BMI x DHI-fu	0.050	0.720
BMI x DHI-em	0.008	0.979
BMI Ix DHI-fis	−0.075	0.588
BMI x DHI-total	0.056	0.685
ACT X VAS	−0.126	0.428
ACT x DHI-fu	−0.061	0.659
ACT x DHI-em	-0.042	0.761
ACT x DHI-fis	−0.008	0.952
ACT x DHI-total	-0.043	0.756
MM X VAS	−0.126	0.426
MM x DHI-fu	−0.021	0.882
MM x DHI-em	0.026	0.852
MM x DHI-fis	0.058	0.679
MM x DHI-total	0.018	0.895
MLG X VAS	−0.110	0.486
MLG x DHI-fu	−0.046	0.739
MLG x DHI-em	−0.039	0.778
MLG x DHI-fis	−0.002	0.987
MLG x DHI-total	−0.035	0.803
MME x VAS	−0.140	0.376
MME x DHI-fu	−0.055	0.695
MME x DHI-em	−0.037	0.792
MME x DHI-fis	−0.008	0.956
MME x DHI-total	−0.041	0.768
MG x VAS	0.299	0.054
MG x DHI-fu	0.080	0.564
MG x DHI-em	0.055	0.691
MG x DHI-fis	−0.033	0.943
MG x DHI-total	0.081	0.561
% GORD x VAS	0.300	0.054
% GORD x DHI-fu	0.065	0.643
% GORD x DHI-em	0.060	0.665
% GORD x DHI-fis	−0.038	0.785
% GORD x DHI-total	0.069	0.620

Abbreviations: % FAT percentage of fat; ACT, total body water; BMI, body mass index; DHI, Dizziness Handicap Inventory; DHI-em, DHI – emotional domain; DHI-fis, DHI – physical domain; DHI-fu, DHI – functional domain) DHI-total, total sum of DHI, FFM, fat free mass; MG fat mass; MM, lean mass; MME, skeletal muscle mass; VAS, visual analog scale.

## Discussion

The present study aimed to analyze a possible association between complaints of vertigo and body composition in post-COVID-19 patients. According to the data presented, we found that there was a significant difference in the vertigo group, with a lower amount of total body water as well as lean, fat-free, and musculoskeletal mass. There was also a significant increase in people who had a feeling of vertigo during and/or after the severe form of COVID-19, with the prevalence of self-reported vertigo increasing from 11.4% before the diagnosis of COVID-19 to 40% of people with vertigo-related complaints during or after COVID-19. The high prevalence is due to the COVID-19 infection and its sequelae. Probably because the pathophysiology of vertigo after COVID-19 is similar to that of other viral infections, with some of its specificities, such as the induction of hypercoagulation and microthrombus formation, which can cause significant circulatory disturbances, possibly affecting its pathogenesis. 16 However, there are still several gaps in the understanding and consensus on the clinical management of these cases.

Severe acute respiratory syndrome coronavirus 2 can also lead to vestibular neuritis causing vertigo and other related symptoms. 17 Furthermore, this high prevalence is in line with the literature in the field, which has shown a significant increase in the prevalence of vertigo in people who contracted COVID-19. 17 18 19 Another factor that may have exacerbated the vertigo complaint refers to the psychophysical stress after an acute infection and mandatory quarantine. 11 18

Considering the aforementioned aspects, muscle mass is a predictor of relevant clinical outcomes in critically ill patients, but in patients hospitalized with COVID-19 and post COVID, this remains to be determined. In view of this, a prospective observational study investigated whether muscle strength or mass would be predictive of length of hospital stay in 196 patients with COVID-19, indicating that strength and muscle mass assessed at hospital admission are predictors of length of stay hospitalization in patients with moderate-to-severe COVID-19. 19 20 In this study, musculoskeletal mass has also proved to be associated with vertigo. Therefore, vertigo and musculoskeletal mass should be considered when treating post-COVID-19 patients. Such findings will certainly provide subsidies for the performance of health professionals in the management of vertigo symptoms, as well as for changes in body composition.

In the comparative analysis of the present research, the data showed difference between the groups without vertigo and vertigo for height and body weight, but not for BMI. This disagrees with the literature in the area, which shows a relationship between vertigo and changes in BMI. 21 22 However, a significant difference was found between the groups with and without vertigo for: body water and lean, fat-free, and skeletal muscle mass. That is, the less body water, lean and musculoskeletal mass, the greater the complaint of vertigo. Although low musculoskeletal mass or sarcopenia has already been associated with vertigo, 23 there are still few studies showing this association in post-COVID-19 patients. The current study brings interesting results but has some limitations that must be considered. A possible limitation is the fact that the vertigo complaints were self-reported, which risks recall bias, in addition to the cross-sectional study design, which does not allow for a cause-and-effect conclusion. However, the questionnaire and scale (DHI and VAS) with categorical variables have been reported to provide superior validity compared to the available answer mode for the dizziness level. In addition, only a few studies that relate the variables vertigo and body composition were found. This may have made it difficult to compare the findings. In light of this, further studies should be encouraged to deepen the knowledge about musculoskeletal mass and their actual differential in vertigo complaints.

Furthermore, balance involves the complex integration and coordination of multiple body systems, including the vestibular, visual, auditory, and motor systems. Balance is guaranteed through the action of the postural control system, with integration of the three sensorimotor subsystems: visual, proprioceptive, and vestibular. These sensory subsystems capture information from the external environment to be sent to the central nervous system, which processes, integrates, plans, and generates a motor response of adequate postural adjustment through the action of the neuromuscular system. Biomechanically, balance requires the maintenance of the center of gravity within the base of support during static and dynamic situations of human movement. Previous studies have found an association between hip and ankle muscle fatigue on unipedal postural control and sensorineural hearing loss. 24 25 Additionally, poor habits such as lack of regular physical activity, low level of physical fitness, insufficient hours of sleep, COVID-19, and nutritional disorders are risk factors for several metabolic and circulatory changes that cause various symptoms, such as dizziness and vertigo. 26 27 28 29

The strengths of this study are the post-COVID cohort's data on several lifestyle-related factors assessed by accurate information on the length of hospital stay and standardized methods.

In this study, the prevalence of self-reported vertigo was 51.4%. These results disagree with the literature in which types of dizziness, is approximately 22.9%. 30 31 This high prevalence was probably due to COVID-19 infection and its sequelae, since only 9.8% of people experienced dizziness prior to the diagnosis of COVID-19. Nonetheless, 40% of patients experienced vertigo during or after COVID-19 infection, and 37.2% reported that vertigo persisted after medical discharge. Therefore, the prevalence of dizziness increased significantly in the studied population after COVID-19. Thus, it can be observed that body composition acts as a factor of impact on vertigo. It can also be an additional factor among the various symptoms related to long COVID.

A thorough history and examination will often provide a clear direction as to the diagnosis, and a correct diagnosis allows treatment for many of the peripheral and central vestibular disorders. 24 Vertigo continues to be an important contributing factor to morbidity and mortality, so it is very important to check the causes and factors associated with it. 26 Volume and intensity of exercise prescription may probably help with balance improvement due to correction of body composition and the use of additional sensory information in post-COVID-19 patients, but more studies are necessary to confirm these findings. The current research also provides a possible explanation to elucidate the contribution of body composition to the postural control system and balance after COVID-19. Thus, the change in people's body composition reduces balance. 32 33 This would be a possible explanation for the findings of the present study, with post-COVID-19 patients with inadequate body composition presenting vertigo. Furthermore, studies are warranted regarding the changes in body composition involved in the physiopathology of dizziness and types of vertigo in population with long COVID.

We also observed an association between vertigo and gender, with women presenting a higher percentage of vertigo. These data agree with those of another article that evaluated 252 patients with chronic dizziness and found statistically significant differences for female patients. 34 An association was also observed between vertigo and the feeling of restful sleep and fatigue; the group with vertigo reported non-restorative sleep more frequently. These data are in line with those of another article that evaluated 96 schoolteachers and found a moderate correlation between total time in bed, sleep efficiency, and DHI, demonstrating that sleep quality should be considered an important factor in the assessment and rehabilitation process of dizziness. 26

Finally, an association was also detected between vertigo and length of hospital stay; These data differ from those of another article that detected an association between needing hospitalization and other symptoms among post- COVID-19 patients. 35

Health promotion programs and health interventions are needed to support people with long COVID. The aspects listed in the present study reinforce the importance of verifying the sensation of vertigo and other vestibulocochlear symptoms as well as body composition in post- COVID-19 patients. These data can serve as a basis for clinical conduct and health promotion to support people with long COVID and vertigo in this direction.

The clinical implications and prospects of this study suggest that body composition should be considered an important factor in the assessment and rehabilitation process of vertigo in after the post-COVID-19 population and in other people with changes in body composition.

## Conclusion

There was a substantial impact of fat-free, lean, and musculoskeletal mass on the sensation of vertigo in patients following the severe form of COVID-19. Therefore, the assessment of body composition should be considered as an important factor in the assessment and rehabilitation of vertigo in this population. Given the above, it is believed that population studies on vertigo and associated factors, involving the different symptoms caused by COVID-19, can contribute to the construction of knowledge about long COVID. Further studies are needed to confirm these findings.
